# Beyond solute clearance: the kidney–gut–immune–nutrition axis in hemodialysis and peritoneal dialysis and a proposed framework for integrated risk stratification

**DOI:** 10.3389/fnut.2026.1931760

**Published:** 2026-10-21

**Authors:** Atul Rawat, Narayan Prasad, Pratima Rawat, Sultan Ahmed, Brijendra Kumar, Ananya Singh, Tanya Nayak, Ravi S. Kushwaha

**Affiliations:** 1 Department of Nephrology, Sanjay Gandhi Postgraduate Institute of Medical Sciences, Lucknow, India; 2 Department of Microbiology, Sanjay Gandhi Postgraduate Institute of Medical Sciences, Lucknow, India

**Keywords:** dialysis, gut, immune, kidney, microbiome, nutrition

## Abstract

Kidney disease affects approximately ~850 million people globally, with millions dependent on dialysis as life-sustaining renal replacement therapy. While dialysis corrects solute accumulation and fluid overload, it incompletely replicates normal renal physiology and introduces its own biological stressors, perpetuating a cycle of systemic dysfunction that extends well beyond the kidney. Central to this dysfunction is the kidney–gut–immune–nutrition (KGIN) axis: a bidirectional network linking renal clearance, intestinal microbial homeostasis, immune regulation, and nutritional metabolism. In end-stage renal disease, each component of this axis is simultaneously disrupted and becomes a driver of further deterioration. Uremic toxin accumulation remodels the gut microbiome toward a dysbiotic state, characterized by depletion of short-chain fatty acid-producing organisms and expansion of pathobionts. The resulting compromise of intestinal barrier integrity permits systemic translocation of lipopolysaccharide and other microbial products, sustaining chronic low-grade inflammation and a paradoxical state of immune dysfunction marked by heightened inflammatory signaling alongside impaired pathogen defense. These perturbations converge on nutritional status, driving protein-energy wasting through catabolic cytokine signaling, anorexia, and suppression of anabolic pathways, with sarcopenia reported in several dialysis cohorts to progress at two to three fold faster than age related muscle loss. Hemodialysis and peritoneal dialysis produce distinct patterns of axis disruption: hemodialysis is characterized by intermittent toxin peaks, bioincompatible membrane-driven complement activation, and more severe dysbiosis, whereas peritoneal dialysis introduces continuous peritoneal glucose exposure and a unique local immune microenvironment, with generally slower decline in residual kidney function. These modality-specific differences translate into divergent cardiovascular, infectious, and nutritional risk profiles that are incompletely explained by traditional clinical variables. Emerging multi-omics approaches, including metagenomics, metabolomics, and high-throughput immune profiling, are beginning to characterize axis disruption with unprecedented resolution, enabling in-depth characterization of host-microbiome-immune identification of patient subgroups with distinct mechanistic signatures that cannot be captured by single domain analysis alone. To bridge this analytical capability with clinical practice, we propose the Microbiome–Immune–Nutrition (MIN) Index, a conceptual, hypothesis generating composite biomarker framework designed to synthesize data across all three axis domains and stratify patients by the severity of disruption to guide targeted, personalized interventions, which remain to be demonstrated through prospective validation studies. Therapeutic strategies targeting the axis, including dietary fiber and plant-based approaches, synbiotics, fecal microbiota transplantation, advanced dialysis modalities, and omega-3 and polyphenol supplementation, show mechanistic promise but require larger, better-designed trials with hard clinical endpoints before routine clinical adoption. Standardization of microbiome research methodology, investment in longitudinal multi-omics cohorts, and development of international collaborative consortia are research priorities that will be essential to translate mechanistic insights into improvements in survival and quality of life for patients on maintenance dialysis.

## 1 Introduction

### 1.1 Global burden of CKD and ESKD

The global epidemiological landscape of renal disease has deteriorated significantly over the past three decades, with contemporary data indicating that approximately 850 million individuals worldwide are affected by kidney dysfunction across all ages and disease stages (1). According to the Global Burden of Diseases, Injuries, and Risk Factors Study (GBD 2023), the global prevalence of adult chronic kidney disease (CKD) among individuals aged 20 years and older has escalated dramatically, rising from 378 million cases in 1990 to 788 million cases (1). This expanding disease burden now accounts for an estimated 1.5 million deaths annually, establishing CKD as the 9th leading cause of mortality worldwide (2). Furthermore, the systemic complications of impaired renal function extend beyond renal mortality, serving as a critical driver of cardiovascular pathology and accounting for 11.5% of all global cardiovascular deaths (1). As the disease progresses to terminal stages, the clinical and economic burden shifts heavily toward kidney failure with replacement therapy (KFRT), or end-stage kidney disease (ESKD) (3). Ageing populations, increased prevalence of diabetes mellitus and hypertension, and environmental conditions, such as exposure to nephrotoxins and dehydration due to climate change influence the transition from CKD to ESKD (4). The GBD 2023 analysis demonstrates that the population requiring life-sustaining renal replacement therapy (RRT) encompassing maintenance dialysis and kidney transplantation has nearly tripled since 1990, reaching 4.59 million individuals (Table 1) (3). However, these prevalence figures expose a critical global “treatment gap” (3). Epidemiological models developed by the International Society of Nephrology (ISN) estimate that between 5.0 and 7.0 million individuals globally require RRT for survival (2, 3). This disparity highlights a profound global health inequity, wherein millions of patients, predominantly in low- and middle-income countries (LMICs), succumb prematurely to KFRT due to restricted healthcare infrastructure and financial barriers (3). Consequently, recorded KFRT prevalence remains heavily concentrated in affluent, high-income nations with robust universal healthcare systems, while notable demographic disparities persist globally, characterized by consistently higher rates of dialysis and transplantation among men compared to women (3).

**Table 1:** Comparison: CKD vs. ESKD/KFRT.

Metric (1, 2, 3, 4, 5)	Chronic kidney disease (CKD)	End-stage kidney disease (ESKD/KFRT)	India/LMIC context
Global population affected	~850 million individuals (All stages)	~4.59 million individuals (Stage 5 on KRT)	Among the highest absolute CKD burdens worldwide; large unmet need for kidney replacement therapy, with most patients requiring dialysis remaining untreated
Primary risk drivers	Type 2 diabetes, high blood pressure, high BMI	Type 2 diabetes and hypertension	Diabetes and hypertension, with additional burden from CKD of unknown aetiology (CKDu) and environmental/nephrotoxin exposure
Highest burden regions	North Africa, Middle East, Central Latin America	High-income GBD regions (e.g., US, Japan, Western Europe)	South Asia a major global contributor; wide inter-state variation in prevalence and access within India
Future outlook	Forecasted to increase by over 30%	Projected financial crisis due to skyrocketing dialysis costs	Dialysis demand rising fastest in LMICs; affordability, access, and infrastructure remain the principal constraints

### 1.2 Dialysis a necessity but biologically disruptive

Dialysis (hemodialysis or peritoneal dialysis) is a life-supporting measure that allows for a degree of renal replacement. It is, however, only a partial physiologic replacement. Although dialysis can effectively clear out small water-soluble uremic toxins and correct electrolyte imbalances, it cannot produce the hormonal, metabolic, and immune effects of normal kidney function (5). In addition, dialysis itself is a biological stressor that can cause systemic perturbations, including bioincompatible membranes, dialysate contamination, vascular access complications, and peritoneal exposure to glucose (6). These disruptions can not only affect kidney clearance but also a wide range of other metabolic, immunological, and nutritional processes. The selection between hemodialysis (HD) and peritoneal dialysis (PD) necessitates a critical appraisal of their distinct pathophysiological mechanisms and systemic biological disruptions. Hemodialysis operates via an intermittent, extracorporeal clearance model that subjects the patient to rapid, episodic fluid and solute fluctuations within tight treatment windows. These aggressive ultrafiltration rates frequently cause intradialytic hypotension and recurrent myocardial stunning, which cumulatively drive chronic myocardial ischemia and systemic vascular calcification (7). Furthermore, the continuous exposure of blood to artificial bio-incompatible membranes and shear stress within the extracorporeal circuit activates complement pathways and circulating leukocytes, inducing a state of chronic, systemic inflammation (8). Conversely, peritoneal dialysis utilizes a continuous, low-flux osmotic model that preserves hemodynamic stability and extends residual kidney function (RKF) significantly longer than HD (9). However, this modality shifts the biological burden toward local tissue remodeling and systemic metabolic dysfunction. The chronic exposure of the peritoneal membrane to hypertonic, acidic dialysates laden with glucose degradation products (GDPs) accelerates the formation of advanced glycation end-products (AGEs), driving submesothelial fibrosis, neoangiogenesis, and eventual ultrafiltration failure (10). Additionally, the constant transperitoneal absorption of dextrose imposes an obligatory systemic caloric load, frequently exacerbating insulin resistance, atherogenic dyslipidemia, and central adiposity (11). Thus, while HD acts as an intermittent, systemic stressor driven by hemodynamic oscillations and extracorporeal bio-incompatibility, PD functions as a continuous metabolic and localized tissue stressor.

### 1.3 Interconnected nature of organ systems

Organ crosstalk refers to complex biological communication and feedback between organs, mediated by mechanical, soluble, and cellular mechanisms, which are essential for maintaining homeostasis but can result in functional and structural dysfunction in other organs when pathological states occur in one or more organs (12). No biological system operates in isolation. Changes in renal function directly alter gut microbial composition by accumulating uremic toxins that serve as selective substrates for pathobiont expansion (13). Altered metabolite profiles from dysbiotic microbiomes impair the integrity of the intestinal barrier, allowing endotoxins to cross and triggering systemic inflammatory pathways (14). The resulting chronic inflammation then creates catabolic states that deplete nutritional reserves, and dietary restrictions used to control uremia further influence microbial ecology (15). Such feedback loops generate a chain reaction of degradation that cannot be solved by addressing any single part of the chain. These complex networks highlight the importance of interorgan interactions for physiological balance and health. New technologies, such as multiomics, AI, and advanced imaging, have accelerated organ cross-talk research, advancing our understanding and potential therapies. The concept of gut kidney crosstalk in kidney disease was first articulated by Andreas et.al (16), and subsequent reviews have elaborated on individual components of this interplay. The present review builds on this foundation in three aspects; 1- it integrates the nutrition and protein energy wasting dimension explicitly alongside the microbiome immune components rather than treating them separately; 2- it systemically contrasts how hemodialysis and peritoneal dialysis differently remodel each axis node; and 3- it translates the resulting system level picture into a composite, prospectively testable risk stratification proposal (the MIN index) rather than restating the mechanistic associations alone. In this narrative review, we synthesize mechanistic and clinical evidence for the KGIN axis across dialysis modalities and propose an integrative risk-stratification framework (Figure 1).

**Figure 1:**
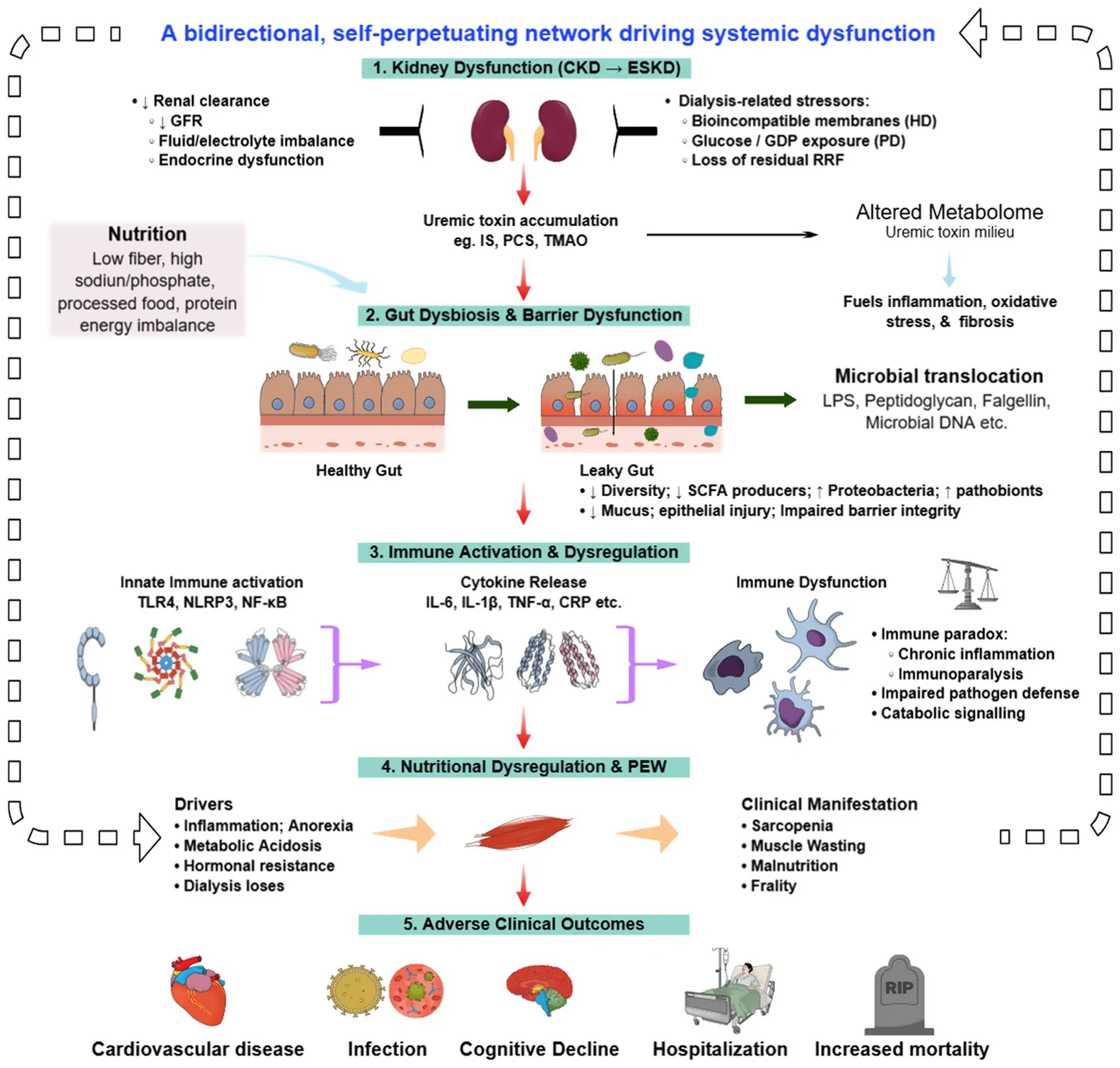
The KidneyGutImmuneNutrition (KGIN) Axis in Dialysis. Progressive kidney dysfunction leads to uremic toxin accumulation, gut dysbiosis, intestinal barrier disruption, immune activation, and protein-energy wasting, creating a self-perpetuating cycle that accelerates systemic inflammation and adverse clinical outcomes. Hemodialysis and peritoneal dialysis introduce distinct biological stressors that differentially influence the KGIN axis.

## 2 Gut microbiome, immune, and nutritional alterations in dialysis

### 2.1 Overview of the kidney–gut–immune–nutrition axis in dialysis

The kidney–gut–immune–nutrition (KGIN) axis describes a multidirectional network in which renal function, gut microbiome composition, immune regulation, and nutritional metabolism are mutually interdependent. Dialysis, while life-sustaining, drives significant remodelling of this axis by altering the composition of the gut microbiota, exacerbating inflammation, and affecting nutrient metabolism. In dialysis-dependent CKD, this axis is disrupted simultaneously at multiple nodes by converging mechanisms that amplify one another rather than operating in isolation. Accumulating nitrogenous waste products alters the gut luminal environment, acting as selective substrates that expand pathobionts and contract beneficial commensals, fundamentally remodelling microbial ecosystem composition and function (17). Dialysis itself introduces additional perturbations layered atop the uremic milieu: hemodialysis membranes activate complement and leukocytes, sustaining a state of bioincompatibility-driven inflammation, while peritoneal dialysis dialysate undergoes glucose degradation, producing advanced glycation end-products (AGEs), thereby imposing a distinct metabolic burden (18). Both modalities compound microbial disruption through dietary restrictions that limit substrate diversity and reduce the generation of fermentation products in the gut lumen. The severity of axis disruption is further modulated by residual kidney function, which declines progressively during dialysis therapy, producing a spectrum of disruption severity even within the same modality (19).

The downstream consequences of this multi-source disruption dysbiosis (imbalanced gut microbiota), elevated uremic toxin burden, systemic inflammation, and nutritional deficiency do not accumulate passively. They form self-reinforcing feedback loops that collectively accelerate cardiovascular risk, immune dysfunction, and mortality (20). Because hemodialysis and peritoneal dialysis generate distinct patterns of axis disruption, the clinical manifestations and the most appropriate therapeutic targets differ meaningfully between modalities, underscoring the need for individualised rather than uniform management strategies (Figure 2; Table 2).

**Figure 2:**
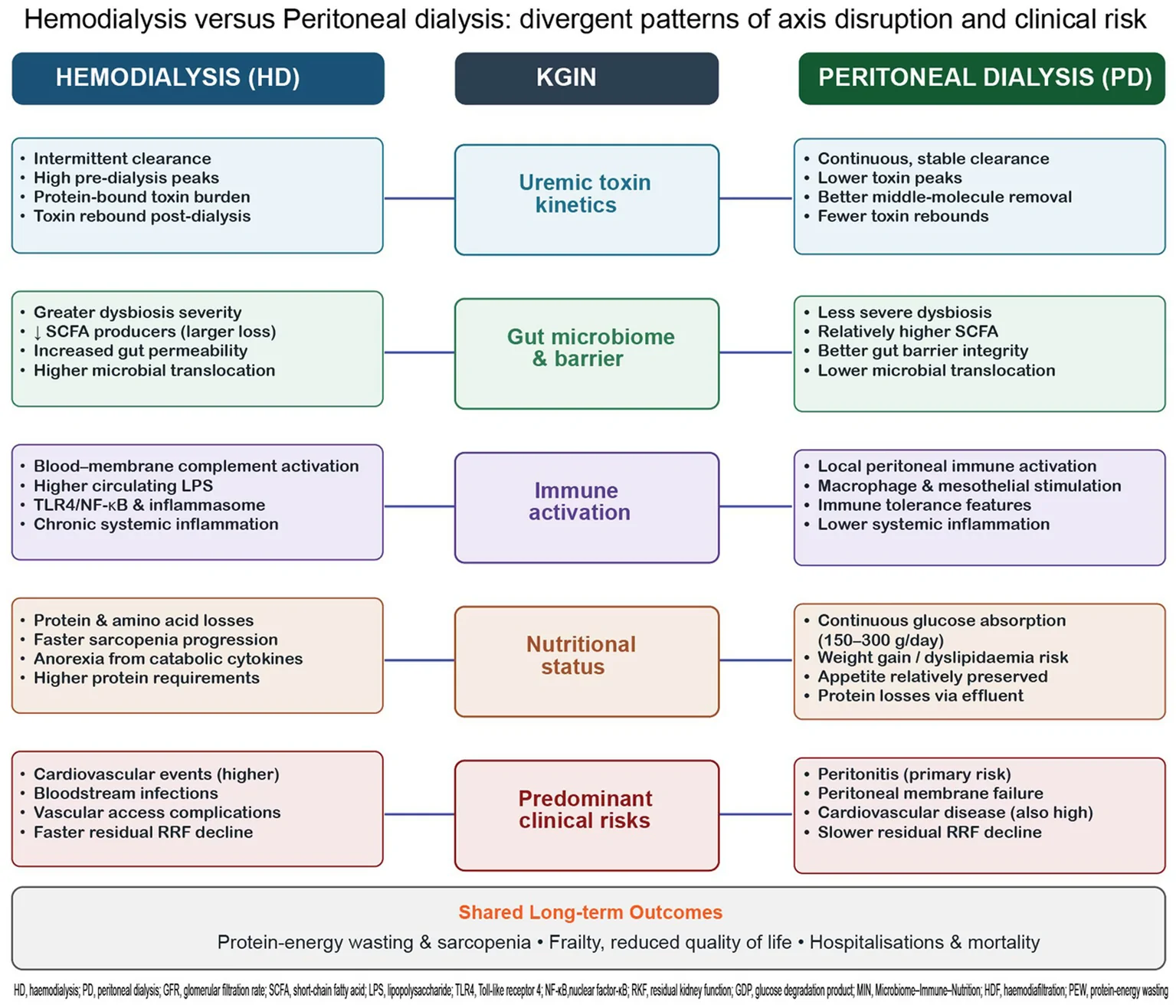
Differential Effects of Hemodialysis and Peritoneal Dialysis on the KGIN Axis. Comparison of the biological effects of hemodialysis (HD) versus peritoneal dialysis (PD). Divergent pattern of axis disruption and clinical risk (cardiovascular events, infection, mortality) across the KGIN domains. HD is characterized by intermittent toxin clearance, complement activation, and greater systemic inflammation, whereas PD is associated with continuous clearance, glucose-mediated metabolic stress, and local peritoneal inflammation. These modality-specific mechanisms produce distinct patterns of gut dysbiosis, immune dysfunction, nutritional alterations, and clinical outcomes while sharing common long-term complications.

**Table 2:** Comparison of hemodialysis and peritoneal dialysis effects on the kidney–gut–immune–nutrition axis.

Parameter	Hemodialysis (HD)	Peritoneal dialysis (PD)
Microbiome	Greater depletion of butyrate producers; Proteobacteria enrichment; more severe dysbiosis overall	Enterobacteriaceae amplification; Bifidobacterium/Lactobacillus depletion; distinct PD-specific profile
Barrier function	Higher LPS translocation; greater endotoxemia; more pronounced tight junction disruption	Moderate barrier dysfunction; peritoneal cavity as additional barrier site
Immune profile	Systemic immune activation; complement activation; bioincompatible membrane effects	Peritoneal mesothelial activation; local and systemic inflammation; preserved RKF benefits
Nutritional status	Higher protein losses through dialysate; more severe PEW; greater amino acid depletion	Continuous glucose absorption; better nutritional preservation early; peritoneal protein losses
Residual function	Rapid RKF decline; greater toxin accumulation; less endocrine preservation	Slower RKF decline; better volume control; preserved erythropoietin longer
Clinical outcomes	Higher cardiovascular mortality; greater infection risk with catheters; fluctuating toxin peaks	Higher peritonitis risk; technique failure with time; better early survival
Dominant min-index domain and priority intervention	Immune–inflammatory dominant; prioritise membrane biocompatibility, enhanced middle-molecule clearance (haemodiafiltration), and anti-inflammatory nutrition (e.g., omega-3)	Nutrition–metabolic dominant; prioritise glucose-sparing/biocompatible fluids, residual kidney function preservation, and protein repletion

### 2.2 Uremia, gut dysbiosis, barrier dysfunction, and metabolomic remodelling

Dialysis modalities profoundly reshape the gut microbial ecosystem, with both HD and PD inducing distinct but equally detrimental patterns of dysbiosis. The universal consequences include reduced microbial diversity and enrichment of bacterial taxa that promote uremic toxin production, though the specific microbial shifts differ between modalities. In HD, characteristic changes include enrichment of Proteobacteria and Fusobacteria, accompanied by depletion of Firmicutes, Actinobacteria, Bacteroidetes, and crucial short-chain fatty acid (SCFA)-producing genera such as Faecalibacterium and Roseburia. Several studies in ESRD and hemodialysis populations have demonstrated that only a subset of patients exhibits detectable circulating bacterial DNA, which is associated with higher CRP and IL-6 levels, implicating systemic microbial translocation in the chronic inflammatory state seen in dialysis (21, 22). As kidney function declines, accumulating uremic toxins fundamentally remodel the gut microbiome and progressively dismantle intestinal barrier integrity, establishing a self-reinforcing cycle of systemic dysfunction. Protein-bound toxins indoxyl sulphate (IS), p-cresyl sulphate (PCS), and trimethylamine N-oxide (TMAO)- accumulate to plasma concentrations 10- to 50-fold above healthy controls as renal clearance fails, rendering the gut lumen a hostile environment for commensal organisms (23, 24). Metagenomic and 16S rRNA sequencing studies have identified consistent dysbiotic signatures in dialysis populations. At the phylum level, Proteobacteria and Fusobacteria expand while Firmicutes and Actinobacteria contract, and the Firmicutes/Bacteroidetes ratio, a marker of metabolic health is markedly reduced compared with healthy controls, correlating with elevated inflammatory markers and higher cardiovascular risk in prospective cohort data (25, 26). At the family level, Enterobacteriaceae, Pseudomonadaceae, and Enterococcaceae increase while Lachnospiraceae, Ruminococcaceae, and Bifidobacteriaceae decline. These taxonomic shifts carry direct functional consequences: urease-producing bacteria proliferate under uremic conditions, driving ammonia production and pH changes that further favour pathobiont expansion, while tryptophanase expressing species amplify indole production, indole being the principal precursor of IS and a driver of the toxin-dysbiosis feedback loop (24, 27). Butyrate-producing species *Faecalibacterium prausnitzii*, Roseburia spp., and *Eubacterium rectale* are consistently depleted across studies.

These compositional shifts directly compromise intestinal barrier integrity. Uremic toxins target tight junction proteins occludin, claudin-1, and zonula occludens-1 through oxidative stress signaling, physically disrupting the paracellular pathway (26). Indoxyl sulfate plays a particularly important role: by activating the aryl hydrocarbon receptor (AhR) pathway, it generates reactive oxygen species that destabilize epithelial junctions and induce apoptosis. Simultaneously, depletion of butyrate-producing bacteria removes the principal energy substrate of colonocytes, weakening the physical barrier and downregulating anti-inflammatory signalling through GPR41 and GPR43 (28). Barrier compromise permits systemic translocation of lipopolysaccharide (LPS), the outer membrane component of Gram-negative bacteria. In health, portal circulation delivers LPS to hepatic Kupffer cells for clearance; in ESKD, reduced hepatic perfusion and impaired clearance capacity allow systemic LPS to accumulate (29). Circulating LPS levels correlate positively with inflammatory markers and negatively with residual renal function, thereby sustaining a state of chronic immune activation (30, 31).

The resulting metabolomics signature distinguishes ESKD from other chronic diseases and reflects the combined impact of dysbiosis and reduced renal clearance. Short-chain fatty acids such as butyrate, propionate, and acetate are significantly depleted in both hemodialysis and peritoneal dialysis patients, compounding the loss of epithelial integrity and GPCR-mediated anti-inflammatory signalling (32). Tryptophan metabolism shifts away from AhR-activating indole production toward kynurenine pathway products, perturbing immune regulatory signalling in ways that may contribute to the immune dysfunction characteristic of uraemia (32). Secondary bile acid synthesis is also impaired, owing to reduced activity of 7α-dehydroxylating bacteria, further disrupting intestinal immune homeostasis (Table 3).

**Table 3:** Key microbial metabolites in dialysis patients: sources, pathological roles, and therapeutic targets.

Metabolite	Bacterial source	Pathological role in ESRD	Therapeutic target	Min Index domain
Indoxyl sulfate (IS)	Tryptophanase-expressing bacteria (*Escherichia coli*, *Proteus*)	Cardiotoxicity; endothelial dysfunction; barrier disruption; AhR activation	AST-120 adsorbent; dietary tryptophan restriction; probiotics	Microbiome → Immune
p-Cresyl sulfate (PCS)	Tyrosine/phenylalanine metabolism (*Clostridium*, *Bacillus*)	Vascular calcification; inflammation; protein-bound toxin accumulation	AST-120; dietary protein modification; synbiotics	Microbiome → Immune
Trimethylamine N-oxide (TMAO)	Choline/carnitine metabolism (*Proteus*, *Providencia*)	Atherosclerosis; macrophage foam cell formation; platelet activation	Dietary choline restriction; carnitine management; TMA lyase inhibitors	Microbiome → Immune
Short-chain fatty acids (SCFAs)	Fiber fermentation (*Faecalibacterium*, *Roseburia*)	Reduced in ESRD; loss of anti-inflammatory signaling; epithelial energy deficit	Dietary fiber supplementation; prebiotics; butyrate enemas	Microbiome→Nutrition→Immune
Secondary bile acids	7α-dehydroxylation (*Clostridium*, *Eubacterium*)	Altered lipid metabolism; FXR signaling disruption; glucose dysregulation	Bile acid sequestrants; microbiome restoration	Microbiome →Nutrition
Lipopolysaccharide (LPS)	Gram-negative bacteria outer membrane	Systemic inflammation; TLR4 activation; immune dysregulation	Barrier restoration; endotoxin binders; anti-inflammatory therapy	Immune

MIN-Index domain column indicates the axis compartment each metabolite primarily reports on (microbiome, immune, nutrition), linking the metabolite panel to the proposed composite index.

### 2.3 Immune dysregulation and chronic inflammation

The immune system in ESKD is in a paradoxical state, labelled “immunological paralysis” (31, 33). Patients in this condition are hyperactive and deficient at the same time, predisposing them to inflammatory and infectious complications. Microbial-associated molecular patterns (MAMPs) and translocated endotoxins (LPS) activate several pattern recognition receptors (PRRs), including Toll-like receptor 4 (TLR4), nucleotide-binding oligomerisation domain-containing protein 2 (NOD2), NLRP3, and retinoic acid-inducible gene I (RIG-I), initiating inflammatory signaling cascades. Once activated, these receptors induce signaling via nuclear factor-kappa B (NF-κB) and the mitogen-activated protein kinase (MAPK) pathway. These cascades produce pro-inflammatory cytokines such as tumor necrosis factor alpha (TNF-α), interleukin-6 (IL-6), and interleukin-1 beta (IL-1β). These cytokines are present at all times in dialysis patients, and their concentration is directly related to prognosis (34). Immunoglobulin levels are also suboptimal in people with innate immune dysfunction, thereby compromising the first line of defense against pathogens. This dysfunction manifests as defects in neutrophil phagocytosis, decreased monocyte oxidative burst activity, and impaired dendritic cell antigen presentation (35). Uremia-associated alterations affect diverse immune cell populations, with documented expansion of proinflammatory CD4 + CD28- T cells and CD14++CD16 + monocytes, alongside progressive decline in natural killer (NK) and B cell numbers (36). These changes arise from the combined effects of uremic toxin retention, oxidative stress generation, and epigenetic modifications, creating a state of simultaneous chronic inflammation and immunodeficiency.

This dual dysfunction is the essence of the clinical paradox for ESKD patients. Inflammation-associated conditions such as cardiovascular diseases, malnutrition, and frailty are on the rise. However, there is a higher risk of infections, such as peritonitis, bloodstream infections, and pneumonia. These relationships are captured by the malnutrition–inflammation–atherosclerosis (MIA) syndrome, a clinically applicable framework introduced to explain the paradoxically high rates of cardiovascular mortality in patients with CKD and ESKD (37). The anorexic effects of inflammatory cytokines are mediated by hypothalamic signals. They also stimulate the degradation of muscle protein by activating the ubiquitin-proteasome system. Beyond cytokine-mediated signaling, uremia is increasingly recognized to reshape regulatory and suppressive immune cell compartments in ways that help explain the co-existence of chronic inflammation with impaired pathogen defence. Expansion of myeloid-derived suppressor cells (MDSCs), upregulation of T-cell exhaustion markers such as programmed cell death protein 1 (PD-1) and T-cell immunoglobulin and mucin domain-3 (Tim-3), and altered regulatory T-cell (Treg) plasticity have each been described in uraemic and dialysis populations, and may contribute to functional immune defects that extend beyond what cytokine profiling alone captures. These cellular mechanisms remain less thoroughly characterized in dialysis-specific cohorts than the cytokine and innate-immune abnormalities described above and represent an important area for future immunophenotyping work within the KGIN framework.

### 2.4 Nutritional and metabolic consequences

The intersection of dialysis, gut dysbiosis, and immune activation creates a perfect storm of nutritional challenges. ESKD patients face competing demands: the need for adequate energy and protein intake to prevent sarcopenia versus the need for dietary restrictions to manage electrolyte imbalances. When inflammation, dietary restrictions, and metabolic alterations converge, they can lead to protein-energy wasting (PEW). This is a syndrome of protein loss coupled with energy wasting, experienced by most dialysis patients. This complex balancing act contributes to the high prevalence (18–75%) of malnutrition in this population (38). It has been shown to be independently associated with higher mortality and hospitalization rates, as well as lower quality of life (39). The sarcopenia component of PEW is particularly important. In kidney disease, muscle mass decreases two to three times more rapidly than in normal ageing. Whether gut dysbiosis is itself a driver of this accelerated muscle loss, or whether the association instead reflects reverse causation (malnutrition and physical inactivity independently degrading gut microbial ecology) or shared upstream drivers such as inflammation, remains unresolved in the current literature; the studies summarized in (Table 4) illustrate the range of designs used to address this question and their inherent limitations. Muscle wasting in dialysis patients reflects altered adipokine and myokine profiles, with elevated leptin reducing appetite and compromised adiponectin contributing to inflammation (40). Simultaneously, elevated myokines, including myostatin and IL-6, promote muscle protein degradation while inhibiting synthesis, positioning these molecules as potential biomarkers for nutritional intervention (41).

**Table 4:** Representative studies on the relationship between gut dysbiosis and sarcopenia in chronic kidney disease, illustrating the current mix of observational and mechanistic-review evidence and the resulting limits on causal inference.

Study (Author, Year)	Design/Population	Principal conclusion
Margiotta et al., 2021 (98)	Cross-sectional observational study; 64 older, non-dialysis-dependent CKD patients (eGFR 10–45), stratified as sarcopenic vs. non-sarcopenic	Gut microbiota composition differed by sarcopenia status and correlated with uremic toxins and inflammatory cytokines, but toxin/cytokine levels themselves did not differ significantly between groups - supporting an association between dysbiosis and sarcopenia without establishing causal direction.
Bakinowska et al., 2024 (99)	Narrative mechanistic review	Synthesises inflammation, metabolic/hormonal dysregulation, gut dysbiosis, and microRNA pathways as converging, plausible contributors to CKD-associated sarcopenia; identifies gut dysbiosis as one candidate mechanism among several rather than as an established primary cause.
Quercia et al., 2026 (100)	Narrative review of CKD-sarcopenia hormonal-metabolic mechanisms	Discusses gut microbiota modulation among emerging therapeutic strategies for CKD-associated sarcopenia, implying a hypothesised but not yet interventionally confirmed causal contribution.
Rusu et al., 2026 (101)	Narrative review of the gut-kidney axis	Frames sarcopenia as a downstream systems-level consequence of gut-kidney axis dysfunction, but relies on mechanistic and associative rather than interventional evidence for this specific link.

The nutritional status of dialysis patients directly influences their microbial and immune environments. Dietary restrictions in CKD (low-protein, low-potassium/phosphate) inevitably limit fiber intake, favoring proteolytic bacteria and uremic toxin production while reducing SCFA-generating saccharolytic taxa. This creates a vicious cycle: dysbiosis promotes inflammation, which drives anorexia and muscle catabolism, further worsening nutritional status. In this condition, disruption and remodelling of the muscle gut axis are key mechanisms underlying this accelerated loss (42). There are multiple convergent pathways involved with the function of the muscle-gut axis in ESKD. Uremic toxins directly inhibit muscle protein synthesis by shutting down the mTOR pathway. At the same time, proteolysis mediated by the ubiquitin-proteasome system is activated. Additionally, a signalling pathway involving reduced availability of short-chain fatty acids (SCFAs) is directly linked to improved insulin sensitivity and anabolic effects on muscle protein (43, 44). The Janus kinase-signal transducer and activator of transcription (JAK–STAT) pathway is activated by inflammatory cytokines, including IL-6. This activation triggers muscle atrophy, increases myostatin expression, and alters IGF-1 levels, creating a less favourable muscle environment (45). At the same time, a low-protein diet, often required to control uraemia, reduces the supply of muscle substrate. Furthermore, due to the catabolic stress of dialysis, dialysis itself increases protein requirements that many patients are unable to meet (44). Despite these recognised mechanisms and associations, there are notable gaps in the current evidence base for effective PEW therapies in dialysis patients. Most strategies, such as nutritional supplementation or exercise interventions, have produced inconsistent results in improving clinical outcomes or reversing muscle wasting. There is a lack of large-scale, well-powered randomised controlled trials specifically targeting the mechanisms of PEW, such as interventions aimed at restoring the muscle–gut axis or modulating relevant inflammatory pathways. Additionally, patient stratification strategies to identify those most likely to benefit from targeted therapies are underdeveloped. Addressing these research gaps is essential to optimise treatment approaches and improve prognosis in this highly vulnerable population.

### 2.5 Dialysis modality and residual kidney function effects

Hemodialysis (HD) and peritoneal dialysis (PD) generate distinct patterns of axis disruption. The differences stem from the radically different strategies these modalities use for renal replacement and their biological effects. Typical changes in HD patients include increased translocation of lipopolysaccharide (LPS) and reduced abundance of butyrate-producing taxa. These traits highlight exposure to bioincompatible membranes and discontinuity in the treatment modality (46). The amount of uremic toxins varies widely during intermittent treatment with HD. Pre-dialysis peaks can harm the epithelium, while post-dialysis nadirs can shift microbial communities toward organisms that colonise beneficially without harm (47). Bioincompatible membranes and contaminated dialysate also contribute to complement activation and cytokine production, exacerbating uremic injury. PD patients exhibit distinct axis characteristics due to prolonged peritoneal glucose exposure and inflammation. Glucose degradation products and advanced glycation end-products present in PD dialysate lead to local and systemic inflammation. This modality also alters the unique peritoneal microenvironment (48). PD patients are characterised by a microbiome shift, with increased Enterobacteriaceae and reduced Bifidobacterium and Lactobacillus. The patterns are not similar to those observed in HD and may be explained by the different metabolic and immune environments in continuous peritoneal therapy (CPT) (49). The PD-specific microbiome literature remains considerably thinner than the corresponding HD literature, both in the number of independent cohorts and in sample sizes. Several smaller studies have reported gut microbial alterations in PD patients, including associations with technique failure and peritoneal dialysis-associated peritonitis, but at least one comparison of PD patients with cohabiting household contacts found no significant difference in microbiota composition once diet and shared living environment were accounted for, underscoring how strongly non-modality factors can confound apparent HD-versus-PD differences (50–52). Most published comparisons across modalities are observational and cross-sectional and have not systematically adjusted for age, diabetes status, residual kidney function, concomitant medications (iron, phosphate binders, proton pump inhibitors, antibiotics), or dietary intake, all of which independently shape microbiota composition and inflammatory tone; the modality-specific patterns described above should therefore be interpreted as associative rather than definitively attributable to dialysis technique per se.

Residual kidney function (RKF) is an important moderating factor across both dialysis modalities, helping reduce the severity of axis disruption. When RKF is preserved, patients demonstrate superior toxin clearance, which, in turn, diminishes uremic-driven dysbiosis and inflammation while safeguarding endocrine functions such as erythropoietin synthesis and vitamin D activation (53). Moreover, preserved RKF allows more efficient volume regulation and less haemodynamic stress to which, in the absence of urine production, anuric people are exposed, which can result in cardiovascular complications.

### 2.6 Integrated feedback loops and clinical consequences

Dysbiosis, toxin accumulation, immune activation, and nutritional decline interact in ways that generate self-perpetuating feedback loops. These loops regulate clinical outcomes in dialysis patients and underlie the progressive decline observed in end-stage kidney disease (ESKD), despite satisfactory solute clearance. More specifically, dysbiosis increases toxin production, further damaging the gut barrier and promoting inflammation. Inflammation induces anorexia and catabolism, starving resident microbes of dietary nutrients while generating inflammatory byproducts that selectively fuel pathobionts. Each time the nutritional status worsens, so does immune competence, increasing susceptibility to infection and triggering further immune responses, maintaining the cycle (54). The relationships between these loops explain why midstream interventions (probiotics, anti-inflammatory agents, and nutrition supplements) are often unsuccessful.

## 3 Integrated systems perspective and clinical outcomes

### 3.1 Interaction between microbiome, metabolome, immunity, and nutrition in HD versus PD

The mechanisms by which the microbiome, metabolome, immunity, and nutrition are integrated at the systems level, along with their interactions, are unique in HD and PD, creating modality-specific clinical phenotypes and necessitating specific therapeutic strategies. The major pathway in HD seems to be the microbiome pathway. Significant dysbiosis results in increased levels of toxins, leading to immune pathways and nutritional deterioration. Fluctuations in toxin levels are especially problematic because epithelial, endothelial, and immune cells experience repeated exposure to toxins and subsequent recovery (55). The extracorporeal circuit itself introduces another immune-activating mechanism for the patient via the membrane-blood interface, complement activation, and, sometimes, the recirculation of dialysate contaminants. This results in an enhanced pro-inflammatory environment that exacerbates uremic inflammation. In PD, the peritoneal cavity adds an additional axis. It creates a local inflammatory environment (during dialysis) that contributes to systemic inflammation via mesothelial cell signalling and cytokine release in the peritoneal cavity (56). The rhythmic effect of PD provides increasingly stable metabolic conditions, allowing the maintenance of remnant kidney function and delaying haemodynamic stress. However, continuous exposure to bioincompatible fluids lead to chronic peritoneal damage and technique loss over time. Such differences in the modalities directly affect how patients should be managed.

### 3.2 Multi-omics approaches and personalised axis modulation

Genomics, transcriptomics, metabolomics, and proteomics are starting to define these complex systems in unprecedented detail, uncovering patient subgroups beyond the current classification system. To explore links between distinct immunotypes and disease severity, Wu et al. (57) combined targeted serum metabolomics and deep immune profiling with gut metagenomic sequencing in a case control cohort of CKD patients and non-CKD controls. Rather than defining discrete clinical subgroups, this study showed that microbiome functional profiles - particularly carbohydrate-active enzyme (CAZyme) gene abundance - predicted early-stage CKD more accurately than models built from serum metabolites or immune cell profiling alone, and identified coordinated correlations linking microbiome-encoded metabolic pathways, circulating amino acids, and specific immune cell clusters. These findings illustrate the potential of multi-omic co-occurrence analysis to reveal host-microbiome relationships not visible to single-domain approaches, though the study did not itself derive or validate distinct patient subgroups with defined mechanistic signatures and sample sizes, a distinction worth noting when this literature is invoked in support of clinical stratification tools.

Recent analytical platforms have transformed the characterisation of KGIN axis components to a previously unattainable resolution, shifting the study of host–microbiome interactions in CKD from descriptive taxonomy toward integrated systems biology. Metagenomic sequencing identifies microbial species composition while metatranscriptomics reveals active metabolic pathways, together providing a dynamic picture of microbial function rather than a static snapshot of diversity (58). Metabolomics platforms mass spectrometry and nuclear magnetic resonance spectroscopy quantify circulating and faecal metabolites that serve as biomarkers of disruption severity and therapeutic response (59). Proteomics and lipidomics profile immune signalling molecules and membrane components, while single-cell transcriptomics elucidates immune cell heterogeneity and identifies the specific subsets governing inflammatory signalling (60). Integrated through network analysis, pathway enrichment, and machine learning, these datasets yield multi-omic signatures that predict clinical outcomes more accurately than any single-domain measure (61). A recent systematic review and meta-analysis showed that combined microbiome metabolite signatures predicted cardiovascular events in patients with CKD with greater accuracy than traditional risk factors alone (62).

The clinical value of this approach lies in its capacity to move beyond population-averaged guidelines toward personalised axis modulation grounded in individual patient phenotypes. Composite biomarker scores derived from multi-omics profiling integrating microbiome composition, metabolite signatures, immune cell profiles, and nutritional status can identify whether a patient’s primary axis vulnerability stems from dysbiosis, immune dysregulation, or nutritional deficiency, and direct intervention accordingly (63). Patients with predominant dysbiosis may benefit most from microbiome-targeted therapies; those with high inflammatory burden from targeted anti-inflammatory strategies; those with nutritional vulnerability from phenotype-specific supplementation rather than generic multivitamin protocols. Machine learning algorithms are particularly well suited to defining patient subgroups with distinct disruption patterns across the microbiome, metabolome, and immune compartment, and to predicting intervention outcomes with greater precision than conventional risk scores. Challenges remain in data standardization, algorithm validation, and clinical implementation, but multi-omics-guided precision medicine in nephrology treatment calibrated to individual biological profile rather than population average represents the most credible path toward meaningfully improving outcomes in dialysis-dependent CKD.

### 3.3 Linking biological mechanisms to clinical outcomes

Biological mechanisms of axis disruption relate to clinical outcomes in well-defined pathways linking molecular perturbations to patient morbidity and mortality. Infection risk is associated with several components of immune dysfunction and microbial dysbiosis, such as peritonitis in PD, in which bacterial contamination from the skin or intestinal tract can seep into the peritoneal cavity where immune defences are compromised, and bloodstream infection in HD, which usually stems from a defect in immune defences at the vascular access resulting in inadequate pathogen containment (31, 58). In both instances, there is a relationship between the severity and recurrence of infections and biomarkers of inflammation and nutrition, suggesting that infection outcomes are linked to integrated axis functioning rather than to a single axis’s dysfunction. In dialysis patients, cardiovascular complications involve the cardio-renal-gut axis, a specialized pathway connecting cardiac and renal dysfunction via gut-derived cytokines with detrimental effects on cardiomyocyte function, including direct pro-oxidant effects of uremic toxins, IS, PCS and TMAO on cardiomyocytes, promotion of vascular calcification in hemodialysis patients, and acceleration of atherosclerosis with increased cholesterol accumulation in macrophages by IS and TMAO, which induces cardiac fibrosis via activation of the renin-angiotensin system (64, 65). These effects on the toxin are exacerbated by systemic inflammation, which may promote atherosclerotic plaque instability and hinder endothelial repair, contributing to a heightened cardiovascular risk profile compared to traditional Framingham predictions (66).

The outcomes of hospitalization and mortality are cumulative measures of axis dysfunction that involve all components. In the dialysis population, the malnutrition-inflammation complex microsystem, expressed as malnutrition-inflammation score (MIS), is more predictive of mortality than are traditional cardiovascular risk factors, with each one-point increase in MIS corresponding to a 10–15% increase in the risk of mortality, regardless of age, diabetes, or dialysis vintage (67). Plasma concentrations of protein-bound uremic toxins predict cardiovascular events and all-cause mortality in prospective cohorts, and in particular, the concentrations of PCS and IS are excellent biomarkers for this (68). These associations indicate that axis disruption biomarkers provide information beyond standard clinical indicators, supporting their use as prognostic tools; they can also help identify disease stage and monitor treatments.

### 3.4 Toward an integrated Microbiome Immune Nutrition (MIN) Index for risk stratification and personalised dialysis management

While multi-omics approaches have significantly advanced our understanding of individual components of the kidney–gut–immune–nutrition axis, a critical translational gap remains: there is currently no robust, integrated tool that synthesizes microbiome, immune, and nutritional data to predict clinical outcomes or inform personalized interventions for dialysis patients. This gap is especially important because single-domain biomarkers fail to capture the complex, reciprocal interactions among these systems that determine patient trajectories. The rationale for developing the Microbiome–Immune–Nutrition (MIN) Index stems from the need for an integrative framework that simultaneously accounts for these interconnected domains and reflects the systems-level disruptions characteristic of dialysis-related pathophysiology. As a composite biomarker strategy, the MIN-Index is proposed to stratify patient risk and guide targeted clinical management more effectively than approaches based on isolated metrics. This proposed MIN index is a testable hypothesis rather than a validated dataset, since no such study has been done which integrates the 3 axis of the kidney ‘gut-immune-nutrition’. Unlike established composite scores such as the Malnutrition–Inflammation Score (MIS), which combines nutritional and inflammatory parameters but does not incorporate direct microbial assessment, the MIN-Index explicitly adds a microbiome domain; it is proposed here as a conceptual framework that will require prospective derivation and validation before clinical use. It is important to be explicit about the current evidentiary status of this proposal: no pilot cohort, simulated dataset, or external validation exists for the MIN-Index at the time of writing, and none of its component weightings has been empirically derived. The index should therefore be read as a structured hypothesis intended to guide future prospective work, not as a tool ready for clinical deployment or patient-level risk classification. Whether a microbiome domain adds meaningful predictive value over the existing Malnutrition-Inflammation Score (MIS), which already combines nutritional and inflammatory indices at a fraction of the cost of microbiome profiling, is itself an empirical question the field has not yet answered; formal head-to-head validation, ideally using net reclassification or decision-curve analyses against MIS and against serum albumin or CRP alone, would be needed before the added cost and complexity of microbiome assessment could be justified for routine use. As summarized in (Table 5) how the MIN-Index compares with the MIS, GNRI, and SGA across assessed domains, required inputs, and validation status, underscoring that its own thresholds remain undefined pending prospective derivation.

**Table 5:** Comparative summary of established nutrition/inflammation composite indices and the proposed MIN-Index, contrasting their assessed domains, required inputs, and validation status.

Index	Domain(s) assessed	Required inputs	Typical threshold/Output	Key reference
MIS (Malnutrition-Inflammation Score)	Nutrition + Inflammation (clinical)	10-component structured history and physical exam (weight change, appetite, comorbidity, subcutaneous fat/muscle wasting) plus serum albumin and total iron-binding capacity	Continuous 0–30 scale; each 1-point increase associated with a 10–15% increase in mortality risk	Kalantar-Zadeh et al., 2001 (67)
GNRI (Geriatric Nutritional Risk Index)	Nutrition only	Serum albumin, actual body weight, ideal body weight	<82 = major risk; 82 to <92 = moderate risk; 92 to <=98 = low risk; >98 = no risk	Bouillanne et al., 2005 (102)
SGA (Subjective Global Assessment)	Nutrition only (clinical/functional)	Structured history (weight loss, dietary intake change, gastrointestinal symptoms, functional capacity) plus physical exam (muscle wasting, subcutaneous fat loss, oedema)	Categorical rating: well-nourished/mild-to-moderately malnourished/severely malnourished	Widely validated general clinical tool; not developed or thresholded specifically for CKD or dialysis populations
MIN-Index (proposed in this review)	Microbiome + Immune + Nutrition (integrated)	16S rRNA or shotgun metagenomic profiling, multiplex cytokine/chemokine panel, biochemical + body-composition + functional + dietary nutrition assessment	Not yet defined; requires prospective derivation and external validation	Proposed framework; no validation cohort exists at the time of writing

To facilitate clinical translation, the MIN-Index could be piloted as a prospective study in selected dialysis centers, where periodic collection of stool, blood, and dietary data is integrated into routine patient assessments. Core components such as 16S rRNA sequencing, high-throughput cytokine profiling, and standard nutritional tests can be implemented using existing laboratory infrastructure, while sample-processing workflows and data-reporting pipelines can be aligned with current clinical schedules. Data collected would be analyzed using validated statistical models and machine learning platforms to generate patient-specific MIN-Index scores at defined intervals; because gut microbiome composition can shift within days in response to diet, infection, or antibiotic exposure, the temporal stability and test–retest reproducibility of a single-timepoint microbiome measurement would themselves need to be established before such scores could be used for clinical decision-making, and repeated or averaged sampling strategies may ultimately be required. This process can be incorporated into multidisciplinary team meetings to inform nutrition, microbiome, and immune-modulatory interventions for high-risk patients. Required resources include laboratory capacity for omics assays, bioinformatics support, staff training for sample handling, and electronic health record integration to store and visualize patient scores alongside other clinical data. Early-phase pilot studies can focus on feasibility, workflow optimisation, and initial validation of prognostic accuracy, enabling researchers and clinicians to envision stepwise scaling and integration of the MIN-Index into clinical practice.

This framework recognizes that Continuous Ambulatory Peritoneal Dialysis (CAPD/PD) and Hemodialysis (HD) differentially remodel the gut–kidney–immune–nutrition axis due to modality-specific stressors, including high-glucose dialysate exposure and iron supplementation in PD, versus bioincompatible membranes, intermittent hemodynamic shifts, and repeated blood-membrane interactions in HD. These differences produce distinct trajectories of dysbiosis, immune remodeling, and nutritional compromise over time. Longitudinal profiling of these domains can therefore yield a clinically actionable composite score that outperforms traditional single-domain markers (such as serum albumin or CRP alone) in risk stratification.

#### 3.4.1 Core components and construction of the MIN-Index

The MIN-Index integrates three interconnected domains: Composite scoring is only meaningful where the three domains are assessed concurrently in the same patient within a defined sampling window; microbiome, immune, and nutrition data collected at different, unlinked timepoints should not be pooled into a single index value, and any reported MIN-Index score should specify the sampling interval over which its inputs were obtained.

Microbiome Domain: Assessed primarily through 16S rRNA gene sequencing (V3–V4 region) for α- and β-diversity, key taxon abundances (depletion of SCFA-producers such as Faecalibacterium and Roseburia; enrichment of Proteobacteria/Enterobacteriaceae), and inferred functional pathways related to uremic toxin generation, SCFA production, and barrier integrity. We note that 16S rRNA sequencing and shotgun metagenomics are not interchangeable: 16S profiling typically resolves composition to the genus level via a single variable region, whereas shotgun metagenomics identifies species- and strain-level taxa and directly characterises functional gene content. Studies using these two approaches are not directly comparable, and a MIN-Index derivation cohort would need to fix a single sequencing modality (and bioinformatics pipeline) throughout, rather than pooling estimates drawn from both.

Immune Domain: High-throughput multiplex immunoassays (e.g., Luminex bead-based panels or proximity extension assays such as Olink) to quantify dozens to hundreds of cytokines, chemokines, and immune-related proteins (IL-6, TNF-α, CRP, galectin-3, etc.), with targeted ELISA validation. Within this domain we distinguish two categories of marker that are frequently conflated in the microbiome-immune literature: systemic circulating inflammatory markers (IL-6, TNF-alpha, CRP), which reflect overall inflammatory tone but cannot localise its source, and markers of intestinal barrier function specifically - plasma zonulin, lipopolysaccharide-binding protein, intestinal fatty acid-binding protein (I-FABP), which has itself been associated with mortality risk in dialysis patients in small exploratory cohorts, faecal calprotectin, or secretory IgA - which are better positioned to indicate a gut origin for inflammation as opposed to, for example, bioincompatible-membrane or vascular-access-driven inflammation arising from the extracorporeal circuit itself (69). Only the latter category should be interpreted as evidence of KGIN-specific (as opposed to generic uraemic or dialysis-circuit) inflammation. This captures the paradoxical state of chronic inflammation coupled with immunoparalysis. Nutrition Domain: Comprehensive multimodal assessment including biochemical markers (albumin, prealbumin, vitamins B12/D, myostatin, GDF-15), body composition via bioimpedance analysis (BIA), functional tests (handgrip strength, Subjective Global Assessment – SGA), and dietary recall for protein, fiber, and micronutrient intake. Food diaries and food-frequency questionnaires are subject to substantial recall and reporting bias, particularly in a population with competing dietary restrictions to track; this inherent imprecision should be reported as a source of measurement error in the Nutrition Domain rather than treated as equivalent in reliability to the biochemical and functional measures in the same domain.

Data integration employs multivariate statistical methods such as partial least squares discriminant analysis (PLS-DA via mixOmics), principal component analysis (PCA), or supervised machine learning (e.g., Random Forests). Features are weighted based on their longitudinal association with hard clinical endpoints, including peritonitis (particularly in CAPD), bloodstream infections and hospitalisations (HD), technique survival, vascular access failure, and quality of life (SF-36). The resulting MIN-Index stratifies patients into low-, moderate-, and high-risk categories, with modality-specific weighting and actionable cut-offs to support clinical decision-making (e.g., intensified nutritional support, targeted synbiotic regimens, or dialysis prescription optimization) (Figure 3).

**Figure 3:**
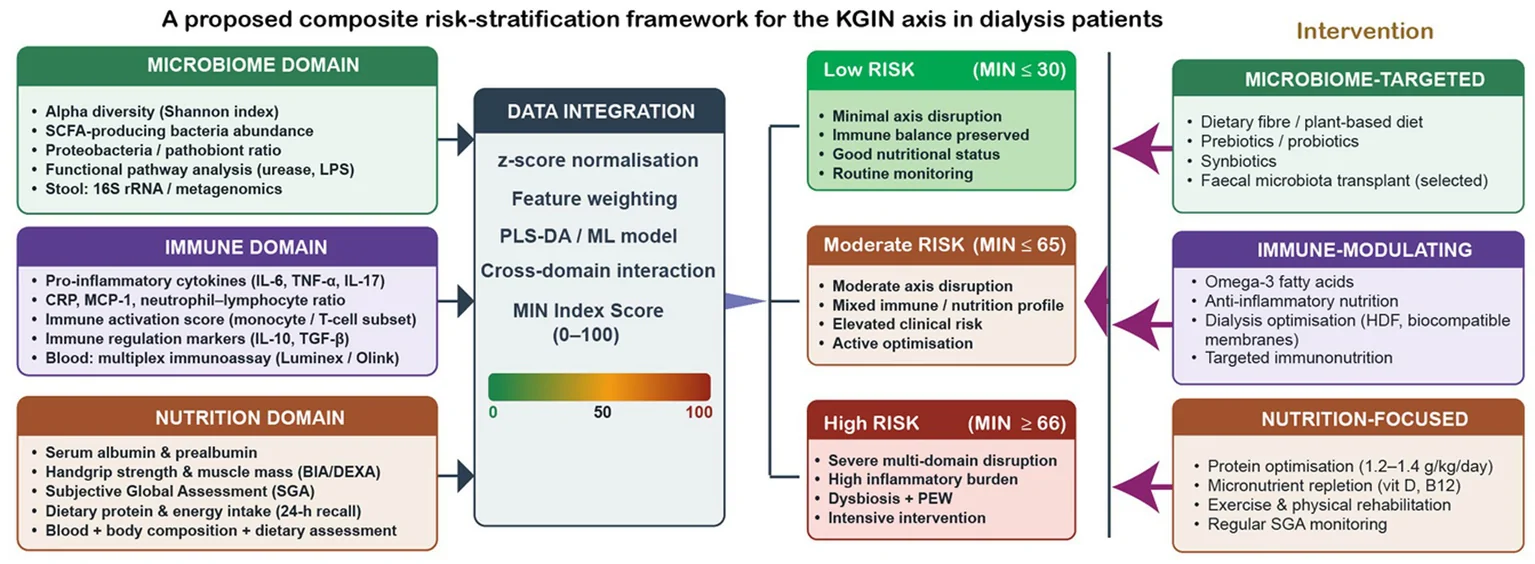
The MicrobiomeImmuneNutrition (MIN) Index for Risk Stratification in Dialysis.Conceptual framework of the proposed MicrobiomeImmuneNutrition (MIN) Index integrating microbiome composition, immune profiling, and nutritional assessment into a composite risk score. The MIN Index classifies patients into low-, intermediate-, and high-risk groups to support personalized interventions, longitudinal monitoring, and precision management of dialysis-dependent chronic kidney disease.

Realizing this scheme requires a dedicated prospective derivation cohort, since PLS-DA and Random Forest weighting cannot be estimated without labelled outcome data. Based on comparable multi-omic biomarker development programmes in nephrology, a derivation cohort of at least 300–500 dialysis patients (encompassing both HD and PD), followed for a minimum of 12–24 months with serial sampling and adjudicated hard endpoints (hospitalisation, peritonitis or bloodstream infection, technique failure, cardiovascular events, and mortality), would likely be the minimum needed to derive stable component weights and subsequently to externally validate them in an independent cohort; smaller or shorter studies would be better suited to feasibility and pilot-phase work than to definitive weight derivation. Three further design requirements follow directly from this. First, concomitant medication data - phosphate binders, potassium-exchange resins, proton pump inhibitors, iron (oral or intravenous), and antibiotic exposure - must be captured and adjusted for as covariates, since these agents can alter gut microbiota composition and mucosal barrier function to a degree comparable to, or exceeding, that of uraemic toxins themselves; without this adjustment the index risks reflecting a patient’s medication burden rather than the severity of axis disruption per se. Second, because dietary prescriptions differ substantially between HD and PD (target protein intake, potassium restriction, and the obligatory caloric load from peritoneal glucose absorption in PD), baseline nutritional intake must be standardised or explicitly modelled in any cross-modality derivation, or the index will simply capture prescribed dietary differences between modalities rather than the pathophysiological impact of dialysis technique itself. Third, because multi-omic profiling generates many thousands of correlated variables per patient, any weighting model is at material risk of overfitting unless the sample size is large relative to the number of candidate features; we therefore propose a strict split-sample design in which weights are derived in one cohort and then tested, without refitting, in a wholly independent external validation cohort, consistent with recommended practice for high-dimensional multi-omic biomarker development (70). Ultimately, clinicians are unlikely to adopt an index that demonstrates only laboratory-level associations: validation should therefore explicitly correlate MIN-Index scores with hard clinical endpoints (mortality, cardiovascular events, infection-related hospitalisation) and with functional status (e.g., handgrip strength), not with biomarker abnormalities alone.

#### 3.4.2 Rationale and anticipated clinical utility

The strength of the MIN-Index lies in its systems-level perspective, moving beyond siloed analyses to reveal mechanistic linkages such as the correlation between loss of SCFA-producing taxa, elevated IL-6/TNF-α, and accelerated protein-energy wasting that drive adverse outcomes. By generating composite data in diverse populations, including Indian cohorts where diet, genetics, and environmental factors shape the microbiome, this framework addresses geographic and ethnic variability often overlooked in existing literature. We reiterate, however, that existing composite nutritional and inflammatory indices (including the MIS comparator discussed above) have themselves been validated predominantly in Western cohorts, and that the same dietary exposure can plausibly produce different microbial and metabolic responses across genetically and culturally diverse populations; geographic and ethnic generalizability should therefore be tested explicitly during MIN-Index validation rather than assumed.

Were the MIN-Index to be validated in future prospective work, its implementation would involve integrating regular microbiome, immune, and nutrition assessments into the routine management of dialysis patients, for example during scheduled multidisciplinary reviews. By generating MIN-Index scores at defined intervals, clinicians could move from reactive complication management to a more proactive and personalized care model. High MIN-Index scores would prompt targeted clinical actions, such as initiating early microbiome-modulating interventions (for example, optimising dietary fibre intake, introducing specific probiotic strains, or prescribing synbiotics), intensifying nutritional support, or increasing the frequency of clinical monitoring. These steps may help to reduce infection rates, hospitalisations, and technique failure. Furthermore, the MIN-Index could function as a stratification tool in the design and conduct of future randomized controlled trials focused on axis-targeted therapies, allowing the identification and enrichment of high-risk patient subgroups most likely to benefit from specific interventions.

A further caution applies to the therapeutic implications of a high MIN-Index score. Several of the axis-targeted interventions discussed in Section 4 themselves carry non-trivial evidence or safety gaps: faecal microbiota transplantation carries a non-negligible risk of infection and bacteraemia in immunocompromised or frail dialysis patients; plant-based and fibre-forward diets carry a real risk of hyperkalaemia in advanced CKD if not carefully managed; and advanced dialysis modalities such as high-volume haemodiafiltration or high-cut-off membranes remove protein-bound uraemic toxins only partially. Recommending these specific interventions on the strength of a MIN-Index result before their own evidence base matures would risk substituting one unproven claim for another. Near-term, MIN-Index-guided action is therefore better directed toward safer, more readily implementable measures - synbiotic or prebiotic supplementation, dialysis-prescription optimisation, and plant-based dietary counselling paired with modern potassium binders (e.g., patiromer or sodium zirconium cyclosilicate) - reserving higher-risk interventions such as FMT for formal trial settings with appropriate safety monitoring.

## 4 Therapeutic strategies targeting the axis

### 4.1 Dietary strategies: fibre-rich and plant-based diets

Dietary interventions are the most readily available and cost-effective means of modulating the kidney–gut–immune–nutrition axis. Practical dietary advice for dialysis patients typically centers on increasing fiber intake, preferably by incorporating fiber-fortified foods or a greater proportion of plant-based meals. These strategies promote the growth of short-chain fatty acid (SCFA)–producing bacteria, thereby supporting gut barrier integrity, enhancing immune regulation, and reducing inflammation (44). For example, patients may be encouraged to add oats, barley, legumes, or low-potassium vegetables to their diet, while simultaneously reducing processed foods that are low in fiber. Plant-based diets confer further benefits by restricting dietary intake of animal-based proteins and processed foods that serve as precursors for uremic toxins and by providing antioxidant phytochemicals to counter oxidative stress (71). The KDOQI 2020 guidelines recommend a daily intake of 25–30 grams of dietary fiber for patients with CKD, yet some high-fiber foods must be avoided or limited due to potassium or phosphorus restrictions. In practice, patients should tailor fiber intake based on specific laboratory values and in consultation with a renal dietitian, who can help identify safe, high-fiber food options within these limits. While mechanistic data are promising, robust evidence from trials on survival or hospitalizations remains lacking, and most studies have focused on surrogate markers such as uremic toxins or inflammatory profiles. Newer dietary strategies, including the use of prebiotic supplements and resistant starch sources, show potential but require further clinical validation in dialysis populations. Cultural and regional dietary patterns further shape how fibre-based strategies can be implemented: in predominantly vegetarian populations, such as much of the Indian dialysis population, baseline fibre and plant-food intake is often already high, and axis-directed dietary counselling may need to focus less on introducing plant foods per se and more on optimizing fiber diversity within potassium and phosphorus limits, while also considering the phytate- and oxalate-related trade-offs of specific plant staples.

### 4.2 Optimisation of protein intake in dialysis patients

Protein intake optimization in dialysis represents a delicate balance between providing adequate substrate for muscle maintenance and limiting the generation of uremic toxin precursors that accelerate axis disruption. The KDOQI guidelines recommend 1.0–1.2 g/kg/day protein intake for dialysis patients, higher than the 0.6–0.8 g/kg/day recommended for pre-dialysis CKD, acknowledging the catabolic stress of dialysis and the protein losses through the dialysate that typically range from 5 to 15 g per day, with higher losses during episodes of peritonitis (72, 73). A high-protein diet, however, also increases uremic toxin production, which could more rapidly disrupt the axis by providing additional substrate for toxin-producing microorganisms. Very low protein diets with ketoanalogues create a metabolic compromise: a significant reduction in nitrogen load and the provision of essential amino acids by nitrogen-free ketoanalogues, which utilize waste nitrogen in transamination while reducing the production of uremic toxins (74). Evidence for the effects of a ketoanalogue-supplemented vegetarian diet on slowing the progression of CKD is demonstrated in clinical trials, but the applicability of these data to ESKD remains unclear, particularly for dialysis patients (75). There is a nuanced, complex logic to how much protein to recommend across the various axis states, wherein patients who have high loads of toxins and are in severe dysbiosis may benefit from more conservative approaches to increasing protein, whereas patients with predominant wasting may require higher levels of protein, even if there are metabolic consequences. In practice, this individualization is best operationalized through serial assessment of nutritional and inflammatory status (e.g., serum albumin, prealbumin, normalized protein catabolic rate, and Subjective Global Assessment) rather than a fixed population-level protein target, reinforcing the broader argument of this review that single-domain metrics are better used to guide individualized titration than to set uniform prescriptions.

### 4.3 Probiotics, prebiotics, and synbiotics

The use of axis modulation in CKD with microbiome-directed therapies has generated significant interest, but clinical evidence remains mixed and insufficient for widespread use in clinical practice. Multiple randomized control trials have been performed to date using a variety of probiotics with small but encouraging outcomes: Administration of *Bifidobacterium longum* BB536 to dialysis patients has been shown to decrease urea nitrogen and improve bowel habits (more than just simple microbial colonization) (76, 77). Prebiotics, such as resistant starch, fructooligosaccharides, and galactooligosaccharides, are intended to support the growth of beneficial bacteria in a selective manner, and it is proposed that they inhibit the growth of pathogenic bacteria and reduce toxins in a more consistent manner than probiotics alone, with the intestinal toxins being channeled towards less harmful pathways by modulation of microbial metabolic pathways (77, 78). This trial’s small sample size (approximately 30 participants), short follow-up, and lack of hard clinical endpoints limit how far these findings can be generalized, a caveat consistent with the broader trial-quality limitations discussed in Section 5.1. In a randomized, double-blind, placebo-controlled trial, it was demonstrated that multi-strain probiotic supplementation reduced inflammatory markers and oxidative stress in PD patients, but did not examine hard clinical outcomes, including peritonitis rates and mortality (20). However, while synbiotics and prebiotics appear to present some theoretical benefits, the evidence for the use of synbiotics in CKD and for prebiotics and probiotics is not sufficient to support their routine clinical use in this context, citing methodological drawbacks such as small cohort numbers, short follow-up periods, and a heterogeneous population of patients reducing the generalizability of the evidence (79).

### 4.4 Fecal microbiota transplantation as an emerging therapy

Faecal microbiota transplantation (FMT) is the most direct strategy for restoring the microbiome, as it transfers the entire microbial community from a healthy donor to a recipient in a single procedure. Unlike probiotics, which require gradual engraftment, FMT transfers an entire microbial community in a single procedure. Preclinical results in CKD are encouraging: in a non-dialysis CKD rat model, animals transplanted with healthy donor FMT showed decreased serum urea and creatinine levels, improved barrier function, and reduced inflammatory markers compared with CKD rats transplanted with sham FMT (80). Because the gut milieu of dialysis-dependent ESKD differs substantially from that of earlier-stage CKD, these preclinical findings should be regarded as hypothesis-generating for dialysis populations rather than directly generalizable to them. An initial clinical study in CKD patients demonstrated FMT’s ability to stabilize creatinine levels and shift microbiota composition toward more beneficial outcomes, though the long-term impact and persistent changes remain to be determined (81). For people undergoing dialysis, the advantages of FMT are particularly promising, as dysbiosis is more pronounced in these individuals than in those with healthy kidneys. Nevertheless, there are a few limitations to clinical application. Donor screening remains a complex process, preparation protocols are difficult to standardise, the regulatory context varies across regions, and concerns such as infection transmission and metabolic syndrome transfer remain unresolved. Moreover, the uremic environment in the gut may quickly favour the regrowth of harmful bacteria, thereby shortening the benefits of FMT and necessitating repeated treatments. Strategies for matching FMT donors to recipients based on metabolic phenotype and disruption patterns could improve FMT efficacy, but such testing would require widespread adoption before becoming routine.

### 4.5 The contribution of Omega-3 fatty acids and polyphenols

Reversing inflammation with nutrition is an alternative way to modulate the axis through immune regulation, rather than direct microbiome intervention, by targeting inflammation as the core driver of much of the axis’s disruption. Omega-3 polyunsaturated fatty acids (PUFAs), such as eicosapentaenoic acid (EPA) and docosahexaenoic acid (DHA), inhibit the production of pro-inflammatory eicosanoids, promote the production of mediators with anti-inflammatory and pro-resolving properties, and regulate the differentiation of T-cells toward regulatory phenotypes (82). Dialysis patients who take omega-3 s have greater reductions in triglycerides and may have a lower risk of cardiovascular disease; however, effects on inflammation and mortality have been inconsistent across studies (83). The discrepancies could be due to the different doses and different levels of omega-3 at the start of treatment. Polyphenols like resveratrol, quercetin and curcumin provide antioxidant and anti-inflammatory effects by blocking the NF-kB pathway and stimulating Nrf2 (84). They also play a role in modulating the composition of the gut microbiome by fostering the growth of beneficial microflora and potentially inhibiting the activity of pathobionts. However, the bioavailability of dietary polyphenols is limited, and therapeutic potential requires higher intake levels, which may be challenging in dialysis patients, where fluid and dietary restrictions restrict intake of polyphenol-rich foods. To improve polyphenol efficacy, encapsulation and targeted delivery systems may be developed, with data in end-stage renal disease (ESKD) that is still quite young and will require specific trials in those populations.

### 4.6 Advances in dialysis techniques and prescriptions

Technological advances in dialysis create opportunities to reduce axis disruption by improving dialysis efficiency and reducing the biological stress on the system. High-flux membranes and online haemodiafiltration provide greater clearance of middle-molecular-weight toxins, such as inflammatory cytokines, and of some protein-bound uremic toxins (85). The enhanced clearance may decrease inflammatory burden and positively affect axis markers. In the CONVINCE (Convincing effectiveness of high efficiency postdilution online haemodiafiltration) trial, the treatment of high-efficiency postdilution online haemodiafiltration reduced all-cause mortality compared with conventional hemodialysis (HD) (86). Mechanistic reviews of HDF membrane and dialyzer design confirm enhanced clearance of beta-2-microglobulin and inflammatory cytokines relative to conventional HD, suggesting plausible benefits for axis modulation (87). However, CONVINCE and most other HDF trials report all-cause mortality or cardiovascular endpoints rather than direct measures of gut microbiome composition or barrier function, so whether enhanced middle-molecule and cytokine clearance with HDF translates into measurable changes in dysbiosis or intestinal permeability remains an open question that has not yet been directly studied. Neutral pH, glucose degradation product (GDP)-low-permeability PD fluids help preserve residual kidney function and reduce peritoneal inflammation. These fluids are mainly good for long-term PD use and can provide more favourable axis profiles when compared with traditional glucose-based PD fluids (88). Refining dialysis prescriptions, for instance, by extending or increasing frequency of sessions, could yield more stable metabolic conditions and reduce axis variability. Nocturnal HD and short daily HD achieve better phosphate control and smaller interdialytic weight gains, with improved blood pressure management compared with the standard thrice-weekly schedule (88). Importantly, these technological innovations offer the potential for broader impact on patient management and outcomes. For example, enhanced clearance of inflammatory and protein-bound toxins may help mitigate chronic inflammation and slow the progression of protein-energy wasting, while improved stability in metabolic parameters could indirectly support gut barrier integrity and immune regulation. In future practice, integrating these advanced dialysis modalities with multidisciplinary interventions such as personalised nutrition and microbiome-modulating therapies may further amplify their benefits. Ongoing and future clinical trials assessing hard endpoints, such as cardiovascular events and quality of life, alongside microbiome, immune, and nutritional parameters, are essential to establish the extent to which advanced dialysis techniques can be leveraged to holistically improve outcomes across the kidney–gut–immune–nutrition axis.

### 4.7 Drug–microbiome interactions and their influence on treatment outcomes

Common drugs used during dialysis have a profound impact on the composition of the gut microbiome, which can also have adverse effects linked to treatment goals. Microbial ecology may also be affected by phosphate binders (sevelamer and calcium acetate) through shifts in intestinal pH and nutrient availability, potentially altering toxin production (89). Iron supplements, which are often used to treat anaemia, promote the growth of pathogenic Enterobacteriaceae and suppress the growth of beneficial species. This microbial shift may further exacerbate dysbiosis, despite an improvement in haematological markers (90). Effective clinical treatment and prevention with antibiotics can also lead to profound, often long-lasting disruption of the microbiome, which can outweigh treatment benefits in some instances, especially with broad-spectrum antibiotics that indiscriminately wipe out commensal populations. Understanding drug–microbiome relationships is essential to optimising therapeutic results. Sevelamer, beyond its phosphate-binding role, adsorbs bile acids and uremic toxins, which may confer microbiome-related benefits beyond its primary purpose (91). Proton pump inhibitors, on the other hand, are frequently used to protect the stomach in dialysis patients, and their use increases intestinal pH and promotes proliferation of Enterobacteriaceae. This may exacerbate dysbiosis and increase the likelihood of infection. Beyond these agents, ESKD patients typically receive multiple additional concomitant medications with documented, if less extensively characterised, effects on the microbiome or immune system: renin-angiotensin-system blockers and other antihypertensives have been associated with shifts in gut microbial composition in cardiovascular and CKD cohorts; statins exert pleiotropic anti-inflammatory and, in some studies, microbiota-modulating effects independent of lipid lowering; erythropoiesis-stimulating agents (ESAs) act primarily on erythropoiesis but may indirectly influence inflammatory tone through correction of anaemia-related hypoxia; and active vitamin D analogues modulate innate and adaptive immune signalling, including effects on antimicrobial peptide expression and T-cell regulation, with plausible downstream consequences for the intestinal barrier and microbiome that remain comparatively understudied in dialysis populations. Given the high pill burden typical of ESKD care, the cumulative, potentially interacting effects of this full medication regimen on the KGIN axis - rather than any single drug class in isolation - warrant dedicated investigation. Personalising prescriptions to address microbiome costs and consequences may enhance axis outcomes without compromising therapeutic efficacy; however, this would require improvements in methods for assessing individual microbiome status.

## 5 Challenges, controversies, and future directions

### 5.1 Gut dysbiosis in CKD: causality, therapeutic limitations, and the path forward

A central controversy concerns whether gut dysbiosis is a causal driver of CKD progression or a downstream consequence of uraemia and dietary restriction; a distinction with direct implications for therapeutic strategy. Evidence supports both directions: uremic toxin accumulation selects for pathobionts, suggesting dysbiosis follows CKD-related metabolic changes, yet dysbiotic communities themselves produce nephrotoxic metabolites that aggravate kidney injury and sustain systemic inflammation (16). This bidirectionality is not merely a conceptual problem it directly underlies the inconsistent results seen in clinical trials of microbiome-targeted therapies. It is worth being explicit about the hierarchy of evidence underlying these claims. A minority of the cited work provides genuinely causal evidence - germ-free or gnotobiotic animal models, interventional FMT studies, and randomised controlled trials - which can support directional inference. The majority of the literature synthesised in this review, however, is observational or cross-sectional, including large registry- or database-derived analyses (for example, NHANES-based epidemiological studies of inflammation and kidney function), which can establish association but not causal direction. Where this review uses causal language (e.g., dysbiosis “drives” or “causes” a downstream change), this should be understood as a summary of the mechanistic hypothesis being tested by the cited authors rather than as a claim that causality has been definitively established, unless an animal-model or interventional study is specifically cited for that claim.

Despite a sound mechanistic rationale, such trials have yielded disappointing results. Probiotics have produced modest reductions in inflammatory markers and uremic toxin levels, but improvements in clinically meaningful endpoints, such as hospitalisation, cardiovascular events, or mortality, remain largely undemonstrated (92). Prebiotics show more consistent effects on toxin reduction yet similarly lack hard outcome data, likely because they address only one component of a multi-factorial axis disruption. Faecal microbiota transplantation remains experimental, with insufficient long-term safety and durability data to support routine clinical use. These failures reflect both trial design limitations small samples, short duration, heterogeneous populations and the biological reality that single-target interventions are unlikely to overcome the compounded, multi-axis disruption characteristic of end-stage renal disease.

Resolving these challenges requires a more sophisticated research framework. Longitudinal cohort studies commencing in early CKD, with serial microbiome and metabolic phenotyping, are needed to define the temporal relationship between dysbiosis and kidney function decline. Study designs must control adequately for host genetics, immune status, medication use, and dietary patterns (19). Stratified analyses across patient subgroups will be essential to capture heterogeneity in axis disruption and differential responsiveness to intervention. Therapeutically, combination regimens targeting multiple axis components simultaneously, rather than isolated microbiome manipulation, are more likely to produce durable clinical benefit and should be the focus of next-generation trial design. It is also worth noting that the feedback loops emphasised throughout this review are described qualitatively; formal quantitative modelling - for example, systems-dynamics or network-based simulations that specify the strength and directionality of each mechanistic link - has not yet been applied to the KGIN axis. Development of such formal models, building on the multi-omics network approaches described in Section 3.2, represents an important next step in moving from a descriptive to a testable systems framework.

A further, more fundamental limitation applies to any single-score index of axis disruption, including the MIN-Index: microbiome composition and its metabolic output are highly individual, such that an identical nutritional or pharmacological stimulus can plausibly produce different metabolites, and therefore different downstream immune effects, in different patients, depending on host genetics, age, stress, circadian rhythms, and prior antibiotic exposure - factors that are difficult to isolate or weight within a single numerical score. Compounding this, there is currently no scientific consensus on the composition of an “optimal” human gut microbiota; in the absence of such a benchmark, any absolute cut-off proposed for an integrated index is necessarily provisional and population-relative rather than a fixed biological ideal, reinforcing the case (Section 5.5) for population-specific reference ranges rather than universal thresholds.

### 5.2 Protein restriction versus risk of malnutrition

The optimal protein intake for dialysis patients remains controversial, with compelling arguments on both sides of the restriction-versus-adequacy debate. Higher protein intakes (1.2–1.4 g/kg/day) are beneficial for nutritional status and maintenance of muscle mass but lead to greater release of uremic toxins and promote axis disruption through increased availability of microbial substrate (93). On the other hand, restricting protein intake reduces the toxin load but may worsen PEW and sarcopenia, especially in patients with low nutritional reserves. Although the KDOQI guidelines attempt to balance these concerns, they provide moderate recommendations (1.0–1.2 g/kg/day), but these requirements may vary significantly based on RKF, inflammatory status, physical activity level, and dialysis modality. Achieving a personalised nutrition prescription based on nutrition biomarkers and metabolite profiling can help resolve this controversy, as this approach acknowledges axis status and considers patient response to nutrition rather than making blanket recommendations. A comparison of restricted versus liberal protein intake should be investigated in future trials stratified by underlying protein-limited disease status and microbiome composition to identify patient subgroups likely to benefit from either approach.

### 5.3 Lack of standardisation in microbiome research methods

Several challenges limit the comparability of microbiota data across CKD studies, including methodological heterogeneity and the difficulty of summarising and interpreting the literature. There are also significant differences in sampling methods (stool vs. mucosal biopsy), DNA extraction methods, DNA sequencing platforms (16S rRNA vs. shotgun metagenomics), and bioinformatics pipelines among studies, which probably contribute to the observed variability in taxonomic and functional profiles that may have been methodological and not biological in nature (24). The absence of standardised reference ranges for “healthy” microbiome composition in CKD also complicates interpretation, as there is inter-individual variability even amongst healthy controls. Building on the Human Microbiome Project and the International Standards on the Human Microbiome, consortia and other groups worldwide should standardise microbiome research in CKD to facilitate meaningful cross-study comparisons and meta-analysis. To address these limitations, concrete steps for standardisation in CKD microbiome research should be prioritised, including the development of consensus protocols for sample collection and processing, the adoption of uniform metadata reporting standards, and the creation of multicenter, cloud-based data repositories that enable researchers to share raw and processed datasets. International collaborative models, such as working groups or consortia, can facilitate the establishment of these protocols and coordinate inter-laboratory validation exercises. Consensus-driven reference panels for healthy and CKD populations could be curated to enable benchmarking and calibration across studies. If this is not standardised, the field risks producing a “noisy” literature, making it difficult to assemble this into a clinically comprehensible body of evidence.

### 5.4 Implementation barriers: cost, access, and regulatory challenges

Translating multi-omics research and microbiome-targeted therapies from bench to bedside faces two interconnected barriers, economic and regulatory, that together constrain both scientific progress and equitable clinical application. Although sequencing costs have fallen substantially, comprehensive multi-omic analysis encompassing metabolomics and proteomics remains prohibitively expensive for routine clinical use, with per-sample costs running into several hundred dollars (94). This burden falls disproportionately on low- and middle-income countries, precisely where CKD prevalence is rising most rapidly, and dialysis infrastructure is already strained. In India and other LMIC settings, where out-of-pocket healthcare expenditure is common and per-session dialysis reimbursement is already tightly constrained, this per-patient cost is unlikely to be feasible for routine, repeated clinical use outside of research settings in the near term, even if individual assay costs continue to fall. Emerging technologies, such as nanopore sequencing, miniaturised mass spectrometry, and multiplexed assay platforms, hold promise for cost reduction, but realising this potential requires targeted investment, infrastructure development, and the adoption of tiered testing systems that prioritise high-value assays for resource-limited settings. Public–private partnerships and open-source analytical platforms will be essential to prevent precision nephrology from becoming the preserve of wealthy healthcare systems. Beyond per-sample cost, turnaround time and the need for dedicated bioinformatics expertise are practical barriers that are easy to underestimate: 16S rRNA sequencing and multiplex immunoassays commonly require days to weeks from sample collection to an interpretable result in most centers, and interpretation of microbiome sequencing data requires specialized analytical capacity that is not routinely available in dialysis units, even in high-income settings. Because the gut microbiome can shift within days in response to diet, infection, or antibiotic exposure, a single-timepoint measurement may also not reliably represent a patient’s steady-state axis status, raising questions about the reproducibility and clinical actionability of any composite score, including the MIN-Index, that has not itself been tested for test–retest stability. These considerations argue for cautious, stepwise feasibility and reproducibility testing rather than an assumption that multi-omics profiling could be integrated into routine dialysis care in the near future. Regulatory frameworks have not evolved at the pace of scientific development. FMT is classified as biological product in some jurisdictions and as a tissue transplant in others, creating compliance complexities that hinder standardization across treatment centers (95). Engineered probiotics and live biotherapeutic products face lengthy, pharmaceutical-style approval pathways that are ill-suited to their unique mechanisms and safety profiles. Adaptive regulatory designs that incorporate biomarker-based endpoints and post-marketing surveillance programs would better balance patient safety and innovation. International harmonization of regulatory standards would also facilitate participation in multi-center trials and accelerate the clinical development of microbiome-based therapies for CKD.

### 5.5 Variability across patient populations

Results from mainly Western cohorts are not necessarily applicable to other demographic and geographic groups, due to variable patterns of axis disruption. Microbiome signatures differ between pediatric dialysis patients and adults, owing to developmental differences in immune maturation and microbial ecology, which may require age-specific therapeutic approaches (19). Sex differences in immune function and microbiome composition, driven by hormonal and genetic factors, could contribute to axis disruption and therapeutic responsiveness in ways not fully captured in the literature. Geographic divergence is particularly pronounced: Indian dialysis populations exhibit micro-transcriptional signatures consistent with dietary behaviors, environmental exposures, and genetic backgrounds that are quite distinct from those in the West, while sub-Saharan African patients exhibit unique nutritional deficiency signatures and infection risk profiles (96). Dietary pattern is a particularly important source of this divergence: a high prevalence of vegetarianism, generous use of spices and legumes, and relatively high dietary fiber intake relative to many Western cohorts are all recognized to shape gut microbiome composition and short-chain fatty acid output in Indian and other South Asian populations (97), and these baseline differences plausibly modify how uremic toxin generation, dysbiosis, and axis disruption manifest in Indian dialysis patients. Any future validation of the MIN-Index or comparable composite tools intended for use in Indian or other South Asian dialysis populations should therefore incorporate population-specific microbiome reference ranges and dietary covariates rather than relying on cut-offs derived from Western cohorts. Rather than adopting protocols based on research from a single population, clinicians and researchers should develop population-specific protocols.

### 5.6 Future research using longitudinal and multi-omics approaches

Future studies should prioritise longitudinal designs that track development over time from pre-dialysis CKD to dialysis initiation and subsequent changes in dialysis modality, to help distinguish causal from consequential relationships. Current cross-sectional designs do not provide information on the temporal sequences of axis disruption or on whether interventions administered at specific disease phases are more effective than those administered at other phases. Multi-omics integration should extend from correlation analysis to network modelling and causal inference methods that define key regulatory nodes to target in therapy (57). The use of standardized protocols and common data repositories would enable large, far-reaching collaborative analyses to overcome the limitations of small sample sizes and single-center studies. International chronic kidney disease microbiome consortia can be established, similar to existing networks in inflammatory bowel disease and cancer research, to combine expertise and resources among researchers across institutions and advance scientific research.

### 5.7 Potential of AI-driven predictive models in nephrology

AI offers great potential for access to research and clinical applications, but its use requires careful validation and consideration of equity issues. By combining multi-omics data with clinical variables, machine learning can provide more accurate predictions of patient outcomes than traditional clinical scoring systems, enabling risk stratification and guiding treatment decisions (97). Natural language processing can extract actionable information from unstructured data, such as dialysis notes and discharge summaries, and computer vision can assess nutritional status and sarcopenia from imaging without an invasive evaluation. New AI applications, however, require attention to the potential harm caused by algorithmic bias against underrepresented populations, data privacy concerns amid increasingly common healthcare data breaches, and rigorous clinical validation prior to first patient use. Establishing explainable AI models that explain AI’s predictions might enhance clinicians’ trust and encourage adoption in nephrology practice.

## 6 Conclusion

The kidney–gut–immune–nutrition axis represents a conceptual and clinical advance in understanding dialysis-dependent kidney disease. Gut dysbiosis, impaired intestinal barrier integrity, chronic inflammation, uremic toxin accumulation, and protein-energy wasting do not operate independently; they form a self-reinforcing network whose collective disruption drives adverse outcomes across both hemodialysis and peritoneal dialysis. The two modalities produce distinct profiles of axis disruption, with differences in microbiome composition, inflammatory burden, preservation of residual kidney function, and nutritional stress that partly explain the divergent cardiovascular, infectious, and metabolic risks observed clinically. Recognizing these modality-specific distinctions should inform more individualized therapeutic approaches and sharper patient stratification in future research.

The field faces well-defined limitations that must be addressed before mechanistic insights can be translated into clinical practice. Studies are predominantly cross-sectional, single-center, and drawn from Western populations, limiting causal inference, generalisability, and applicability to the demographically diverse global dialysis population. Methodological heterogeneity in sampling protocols, sequencing platforms, and reporting standards undermines comparability across studies. Therapeutic trials have been small, short, and inconsistent in their endpoints. Collectively, these gaps create uncertainty about the direction and clinical magnitude of observed microbiome alterations and leave the therapeutic evidence base preliminary.

Addressing these gaps requires a coordinated research agenda built around five priorities: establishing large-scale longitudinal cohorts with serial microbiome, immune, and nutritional phenotyping to resolve causality; conducting multi-center randomized controlled trials of combination interventions using patient-centered endpoints; validating multi-omics biomarkers, including the proposed MIN Index across ethnically and geographically diverse populations; standardizing pre-analytical and bioinformatics protocols to enable pooled analyses; and investing in international collaborative consortia with open-access data-sharing infrastructure. Progress on these fronts will be essential to translate the mechanistic promise of the KGIN axis into measurable improvements in survival and quality of life for patients on maintenance dialysis.
